# Impacts of racism on First Nations patients' emergency care: results of a thematic analysis of healthcare provider interviews in Alberta, Canada

**DOI:** 10.1186/s12913-022-08129-5

**Published:** 2022-06-21

**Authors:** Patrick McLane, Leslee Mackey, Brian R. Holroyd, Kayla Fitzpatrick, Chyloe Healy, Katherine Rittenbach, Tessy Big Plume, Lea Bill, Anne Bird, Bonnie Healy, Kristopher Janvier, Eunice Louis, Cheryl Barnabe

**Affiliations:** 1 Alberta Health Services, Strategic Clinical Networks, Alberta Health Services Corporate Office, Seventh Street Plaza, 14th Floor, North Tower, 10030 – 107 Street NW, Edmonton, AB T5J 3E4 Canada; 2 Department of Emergency Medicine, University of Alberta, 790 University Terrace Building, 8303 - 112 Street, Edmonton, AB T6G 2T4 Canada; 3 University of Alberta, School of Public Health, Edmonton Clinic Health Academy, 3-341, 11405 87 Ave NW, Edmonton, AB T6G 1C9 Canada; 4 Blackfoot Confederacy Tribal Council, P.O. Box 916, Standoff, AB T0L 1Y0 Canada; 5 University of Calgary, Department of Psychiatry, Health Sciences Centre, 3330 Hospital Drive NW, Calgary, AB T2N 4N1 Canada; 6 University of Alberta, Department of Psychiatry, 1E1 Walter Mackenzie Health Sciences Centre, 8440 112 St NW, Edmonton, AB T6G 2B7 Canada; 7 Stoney Nakoda Tsuut’ina Tribal Council, PO Box 350, 9911 Chiila Boulevard S.W., Tsuut’ina, AB T2W 6H6 Canada; 8 Alberta First Nations Information Governance Centre, Suite 101, 535 8 Ave SE, Calgary, AB T2G 5S9 Canada; 9 Paul First Nation Health Services, Box 89, Duffield, AB TOE 0N0 Canada; 10 Kee Tas Kee Now Health Services, KTC Sub Office, 17015 – 105 Avenue NW, Edmonton, AB T5S 1M5 Canada; 11 Maskwacis Health Services, Box 100, 14 Ermineskin Ave, Maskwacis, AB Canada; 12 University of Calgary, Departments of Medicine and Community Health Sciences, Health Sciences Centre, 3330 Hospital Drive NW, Calgary, AB T2N 4N1 Canada

**Keywords:** Indigenous peoples, First Nations, Indigenous health, Emergency medicine, Racism, Discrimination, Healthcare providers, Health services, Medical education, Cultural safety

## Abstract

**Background:**

First Nations people experience racism in society and in the healthcare system. This study aimed to document emergency care providers’ perspectives on care of First Nations patients. First Nations research partner organizations co-led all aspects of the research.

**Methods:**

Sixteen semi-structured interviews were conducted with Alberta emergency department (ED) physicians and nurses between November 2019 and March 2020.

**Results:**

ED providers reported that First Nations patients are exposed to disrespect through tone and body language, experience overt racism, and may be neglected or not taken seriously. They described impacts of racist stereotypes on patient care, and strategies they took as individuals to address patient barriers to care. Recognized barriers to care included communication, resources, access to primary care and the ED environment itself.

**Conclusions:**

Results may inform the content of anti-racist and anti-colonial pedagogy that is contextually tailored to ED providers, and inform wider systems efforts to counter racism against First Nations members and settler colonialism within healthcare.

**Supplementary Information:**

The online version contains supplementary material available at 10.1186/s12913-022-08129-5.

## Background

The state known as Canada is a colonial state, where a settler population has asserted sovereignty on lands taken over (without legal basis in large parts of the country) from Indigenous nations [1–3]. The term First Nations refers to a large number of distinct Indigenous nations who differ from two other broad categories of Indigenous peoples in Canada, the Inuit and Métis [4]. The role the health system has played in the colonization of Indigenous peoples has been described by Lux [5], and Razack has described cases where settler institutions contribute to the deaths of Indigenous persons despite claiming to fulfill their legal and self-asserted duty of care [2]. Informed by such critical understandings of the role of healthcare within colonialism, this article examines care of First Nations members within emergency departments (EDs). To do so we draw on interviews conducted with ED physicians and nurses in Alberta, Canada.

In Canada, Indigenous peoples have been subject to systematic state sponsored violence as part of processes of land expropriation [1, 3, 6, 7]. Tens of thousands of Indigenous children were removed from their family homes for assimilation within state sponsored residential schools which operated for over 100 years [3]. In these schools adults who should have been caregivers caused the deaths of many of the children through abuse, neglect and allowing the spread of tuberculosis [3]. Within healthcare, twentieth century “Indian” hospitals reproduced many of the harms and assimilationist efforts of residential schools while focusing on isolating contagious diseases (including tuberculosis spread by residential schools [8]) within Indigenous populations and away from settler communities [5].^1^ Both the residential schools and the hospitals functioned to support settler colonialism as they worked “to remove, detain, assimilate and eliminate” Indigenous peoples [8].

Land claims remain unsettled and contested, such that colonization is incomplete and ongoing in Canada today. Colonialism thus remains a contemporary reality of Indigenous peoples in Canada, and a key determinant of Indigenous health [10]. As one contemporary consequence of colonialism, Indigenous life expectancy in Canada is markedly shorter than the Canadian average [11]. In resisting this situation, Indigenous peoples have never relinquished their sovereignty, and continue to contest racism and colonialism within the wider society. Such racism is well documented within healthcare [12–19].

A series of twenty-first century deaths of First Nations patients who were not provided adequate care within the health system have been widely reported and demonstrate the urgency of contesting racism and colonialism. In 2008, Brian Sinclair died of an untreated bladder infection in an ED waiting room in Winnipeg, without seeing a health professional, after being ignored for 34 hours because staff assumed he was intoxicated or homeless [20]. In 2015, Keegan Combes died of poisoning in a British Columbia hospital because he was assumed to be intoxicated and not treated until it was too late [18]. The 2020 death of Joyce Echaquan has garnered national attention and brought anti-Indigenous racism in healthcare to the forefront of national dialogue. Ms. Echaquan was an Atemekw woman who video-recorded and broadcast her calls for help in a Quebec hospital. She died restrained and subject to what has been widely characterized as racist and misogynistic verbal abuse by providers [21]. The investigating coroner has found that systemic racism contributed to her death [22]. To counter systemic racism, Atamekw organizations have proposed “Joyce’s Principle” which would “guarantee to all Indigenous people the right of equitable access, without any discrimination, to all social and health services, as well as the right to enjoy the best possible physical, mental, emotional and spiritual health.” They have called upon the governments of Canada and Quebec to adopt this principle [23].

The cases described above raise questions of how frequently racist treatment occurs, and how often racism results in avoidable morbidity and mortality of First Nations patients, without the cases receiving national attention. First Nations team members note that many more stories of racism in care are well known and shared within their communities. There is a need to document these stories, to combat racism and halt the continuing accumulation of similar cases.

We define racism as a system [24–27] that consists of “structures, policies, practices, and norms resulting in differential access to goods, services, and opportunities of society by ‘race’.” [24]^2^ Such goods and services include healthcare. “Race” is put in quotations, to acknowledge that it is not a natural reality, but the product of socially categorizing individuals based on phenotype, for the purpose of advantaging some by disadvantaging others [24]. Racist ideas have been a key construct in colonial thought and practice in Canada, used by settlers to justify ongoing expropriation of Indigenous lands and harms done to Indigenous people [1, 2, 7]. We define colonialism as “the processes by which Indigenous Peoples were [and are] dispossessed of their lands and resources, subjected to external control, and targeted for assimilation and, in some cases, extermination” [7] together with the rationalizations colonizers offer for these processes [7, 28].

Alongside racism and colonialism, as part of our analytic framework, we employ the term discrimination defined as “differential actions toward others by ‘race’” [24] and stereotypes defined as ideas about what members of groups are like [29]. When stereotypes are applied to ‘racial’ groups they are racist stereotypes. A number of studies have previously reported healthcare provider stereotypes around First Nations patients as substance using [7, 14, 16, 17] and as not taking care of their health [17]. Such racist ideas have obvious potential to negatively impact healthcare. Related literature documents that experiences of anti-Indigenous racism may be intensified in the ED environment [12, 14–16].

Since 2017, our team has been engaged in a mixed methods study of care experienced by First Nations patients in EDs in Alberta. Our previous work documents that 9.4% of emergency visits in Alberta are made by “status” First Nations persons [30], that First Nations members expect discriminatory treatment in the ED in Alberta [16], and that First Nations patients are not triaged (assessed) as needing care as urgently as non-First Nations patients in EDs [31]. In this manuscript, we present results of a thematic analysis of interviews with emergency care providers regarding their perceptions of the care received by First Nations members.

While Indigenous cultures, health and healthcare have been increasingly suggested areas of knowledge for physicians [32, 33] and nurses [34, 35], we approach this topic from the stance that ensuring equitable care of First Nations members requires examining how racism and colonialism function within healthcare. We specifically focus on emergency care because of First Nations team members’ concerns about EDs as a site of racism and literature supporting these concerns [14, 15, 36].

We advance the literature on anti-Indigenous racism within emergency care by providing detailed descriptions of racist stereotypes held by providers in a particular time and place, as expressed by healthcare providers. In turn, we enhance the sociological literature on ED care. This literature documents that ED providers operate in conditions of limited resources and manage the complexity of their work through categorizing patients as examples of recognizable types [37–39]. This categorizing work has been shown to reflect wider societal moral judgments about deserving and undeserving “types” of people, as well as considerations of which patients are presenting “legitimate” emergency cases [38, 39]. Through this categorization work, limited ED resources are rationed out to patients based on perceptions of their need, deservingness and the capacity of the ED to assist them ([38] citing, [40]) This rationing determines how long patients will wait for care, time allocated to each patient by providers, whether (and what) care will be provided within ED and whether a patient will be referred elsewhere [38]. We discuss the ways racial stereotypes of First Nations members interact with ED practices of categorization and rationing, in ways that reinforce colonialism. We argue that recognition of colonialism by providers and provision of appropriate resources within and beyond EDs, are necessary to improve First Nations patients’ emergency care. We also describe individual providers’ self-reported efforts to counter racism and systemic barriers to care for First Nations patients, and build on these to offer related recommendations for providers, EDs and healthcare systems.

## Methods

### Community engagement and ethical data ownership

Our research project was developed in a collaborative process among First Nations partners in Treaties 6, 7 and 8; with two universities and the provincial health authority. Project support, and community and Elder engagement, was facilitated by the Alberta First Nations Information Governance Centre (AFNIGC), Maskwacis Health Services, Yellowhead Tribal Council, Stoney Nakoda Tsuut’ina Tribal Council, the Blackfoot Confederacy, and the Treaty 8 Organization of First Nations of Alberta. AFNIGC is the custodian of the qualitative data on behalf of First Nations people, in compliance with principles of Ownership, Control, Access to and Possession of data (OCAP®) [41, 42]^3^.

### Sampling

We employed theoretical sampling, as we aimed to recruit the most relevant professional groups with experiential knowledge of our topic of study. Furthermore, we aimed for variation within our sample, by attempting to recruit men and women, and persons who had experience in rural, urban and remote emergency care sites. Our sampling framework is provided as Additional file 2: Supplement 1. EDs targeted for recruitment efforts were chosen to reflect distinct facility sizes and types in rural communities and urban centres. Outside Alberta’s two metropolitan centres, rural sites were selected based on proximity to partner Nations and proportions of First Nations patients attending those sites. In our larger study, we have conducted sharing circles with First Nations community members and interviews with First Nations health directors. We report data from provider interviews in this paper.

### Participant recruitment

ED nurses and physicians were recruited for interviews by email through the professional networks of project team members, which includes the Emergency Strategic Clinical Network. Physician leads and managers of specific EDs were also asked to circulate the invitation to their staff and physician email lists. Efforts were made to recruit First Nations physicians and nurses as key informants, through emailed invitations sent directly by team members with professional connections to these providers.

All participants were emailed study information letters and provided the opportunity to ask questions before signing consent forms and returning these by email. Participants completed demographic questionnaires, and received a $25 gift certificate as acknowledgement of their time. Recruitment was halted in March 2020 because we found that ED providers ceased volunteering for interviews during the COVID-19 pandemic.

### Interview content and data collection

Interview questions were developed by PM and LB based on previous research and familiarity with the literature, revised based on the advice of a project-specific Elder Advisory group, and further revised during the course of interviews. The final interview guide is available as Additional file 2: Supplement 2. Interviews were conducted by phone by PM, LM or both, between November 2019 and March 2020. These were recorded and transcribed verbatim.

### Analysis

In results below, physician participant quotations are labeled as “P#,” while nurse quotations are labeled with an “N#.” Thematic analysis was conducted [43, 44]. Each transcript was coded by LM and at least one other team member (PM, CH, KF), both inductively and deductively with a literature-based framework (which relied on [14, 16, 45, 46]) using QSR NVivo 11 software [47]. The coding team met throughout analysis to discuss and refine the coding framework and to maintain reflexivity. This work is informed by Indigenous ways of knowing [48, 49], and aligned with critical Western paradigms [50], in recognizing that research is always a situated activity that is never simply knowledge producing but which necessarily reinforces or challenges existing hierarchies and distributions of resources. Our team brings together expertise in Indigenous ways of knowing, critical social theory, emergency medicine and health services research. The project co-leads are a Traditional Practitioner/Knowledge Keeper (LB) and a PhD Sociologist (PM). BRH is a practicing emergency physician and holds leadership roles within emergency medicine. BH was Executive Director of AFNIGC at time of the project initiation, and oversaw approval of the project by the Assembly of Treaty Chiefs in Alberta. She brings a wealth of experience in First Nations research and data sovereignty, trained as a Registered Nurse with ED experience, and is currently the Health Director of the Blackfoot Confederacy. CB is a Métis physician and health services researcher. Other team members include First Nations health system staff, and further detail may be found in the authors’ information section. Triangulation of our different disciplinary perspectives and lived experiences in analysis of our results has served to enhance the confirmability of our analysis, as we question one another’s predispositions and enhance one another’s knowledge by reflecting distinct worldviews [51].

First Nations team members provided Indigenous perspectives to the analysis and validated results by ensuring that this manuscript reflects their experience as First Nations patients who utilize emergency care and as First Nations members working within the healthcare system. Results were also affirmed as appropriately serving to enhance First Nations’ desires for equitable care by the project Elder Advisory when results were presented to Elders in February 2021.

## Results

Interviews were completed with 11 physicians who provide emergency care and 5 ED nurses. Interviews lasted between 60 and 90 minutes. Despite efforts to recruit First Nations physicians and nurses, all participants identified as non-First Nations. We did not press the First Nations providers who we know work in emergency settings in Alberta to participate, as this is a relatively small number of individuals, and assertive recruitment efforts could easily have become coercive if potential participants felt their participation was required to maintain personal or professional relationships.

Participants reported experience working in 10 distinct hospitals (6 urban and 4 rural), and a median of 11.5 years of work experience in EDs with an interquartile range of 5.75 to 18.75. Other participant demographics are provided in Table 1.

**Table 1:** Demographics

	N	%
**Age**		
20–30	2	13
31–40	7	44
41–50	3	19
51–60	3	19
61–70	1	6
**Sex**		
Male	5	31
Female	11	69
**Role**		
Physician	11	69
Nurse	5	31
**Rural / Urban**		
Rural	4	25
Urban	12	75
**Hospital types participants had experience working in**^**a**^		
Mixed Tertiary Hospital	10	63
Pediatric Tertiary Hospital	4	25
Regional Referral Hospital	1	6
Community Hospital	4	25
**Training on First Nations Health Care**		
Health authority online modules	6	37.5
Health authority modules and other course(s)	8	50
Other course(s)	1	6
No training	1	6
**Experience living/staying in First Nations communities**	3	19
**Experience working in First Nations communities**	5	31

^a^Several participants worked in more than one hospital type

**Fig. 1:**
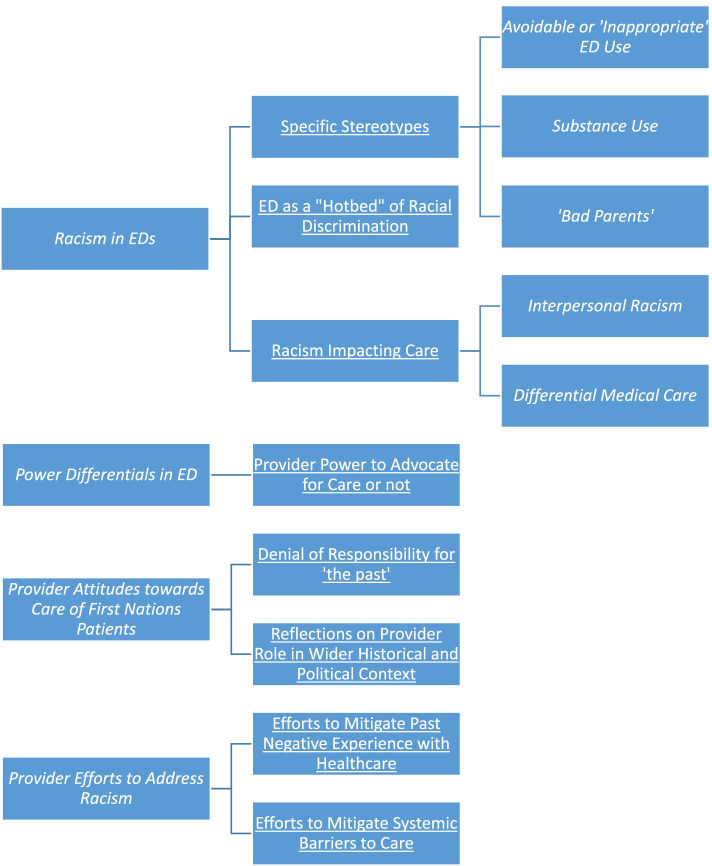
Structure of themes and subthemes

Providers described racism and stereotyping, the way the ED environment interacts with patient care expectations, power differentials, their attitudes, and their efforts to address racism and recognized systemic barriers in providing emergency care to First Nations patients. In addition to the quotations in the text below, we provide additional quotations for each theme in Additional file 1: Appendix. Figure 1 provides a visual overview of our theme structure.

### Racism in EDs

All providers noted that racism and stereotyping occur in EDs. As one provider stated, “I think everything that happens in the regular community, umm, like all of the stereotypes that are common in Alberta also exist within [the] department….” (P17)^4^ The provider gave specific examples of racist stereotypes in the following statement:

“…the idea of coming to the emerg and ‘Oh another Indigenous or First Nations patient with a ton of kids’ or ‘Oh, is [Children’s Services] involved?’ or um, you know, yeah, all of those sorts of things …- they’re not as educated, they are squandering their money from the government. All of those things that are common in society also occur in the emerg.” (P17)

A rural provider felt that providers in the local ED had strong negative opinions of First Nations people from nearby reserves, saying “Their perception is that the First Nations people out here are very dangerous people and you’re not safe out here if you’re white.” (N10).

#### ED as a “hotbed” of racial discrimination

Providers described the ED as a place where racial discrimination comes into play forcefully. “[T]he ED is probably a hotbed for discrimination and unequal care... I think it’s really just because First Nations patients will come to us, as do other people, when they’re at their worst in terms of a crisis.” (P11) Providers further acknowledged that First Nations patients come to ED expecting discriminatory treatment (see Additional file 1: Appendix).

#### Specific stereotypes held by ED providers about First Nations patients

##### Assumption of avoidable or inappropriate ED presentations

Multiple providers expressed that First Nations patients are often assumed to not require ED care. A nurse suggested that assumptions of ED misuse influence perceptions of First Nations patients even before they arrive in ED. “I would say as a general rule, as soon as we hear that there’s an ambulance coming in from [a specific Nation] there’s an assumption that it’s going to be something silly coming in.” (N1) A particular striking example involved the use of a highly derogatory term for First Nations women being linked to perceptions of ED misuse.

“there’s been terminology of like ‘oh they’ve dropped off, or the bus has come in’ with, this kind of a way of saying, that the transport van from a reserve has come in. I’ve heard terms like ‘[Sq-word]-pack’ Um, I’ve heard terms like ‘another Indian is in’, which is very uncomfortable to say out loud… all of a sudden ten people have shown up, probably no one is that sick, and then they use the term [Sq[word]-pack] as an expression of kind of annoyance.” (P17)

##### Substance use

Many providers expressed a perception that First Nations patients are over-represented in ED presentations for substance use disorders, particularly alcohol use disorder.^5^ One provider said,

“I would say fairly commonly on a weekend night, if a patient is coming in with paramedics and they’re in their mid-teens and they come in and the paramedics said, you know, ‘we’re bringing someone in and they’re intoxicated,’ quite often we’re going to say, ‘oh I wonder if they are, I wonder if they’re Indigenous?’ And I’m going to say probably seventy, it sounds bad, but probably seventy percent of the time they are. And then it's kind of like, you kind of think to yourself, ‘oh too bad, I wish that they weren’t.’ Because it reinforces your thinking about that population.” (N5)

##### Bad parents

Multiple providers described a bias or assumption that First Nations parents lacked capacity to care for their children. One provider felt that “maybe one of the strongest assumptions that’s made is that there is a lack of capacity, potentially, amongst First Nations families.” (N2)

Another provider perceived that ED staff “could be like assuming that someone is intentionally not caring about their child, or that whatever is going on with their kid is their fault.” (P4) The provider noted, “I guess there is an unconscious bias that there would be a, that it would be more likely that the child would suffer abuse if they’re Indigenous.” (P4)

A third provider felt that there are assumptions that First Nations women have a lot of children and that Children’s Services is often involved, with the result that First Nations women “might be asked more frequently [than non-First Nations women] like well, do you have kids? Where are the kids?” (P17)

#### Racism impacting care

##### Interpersonal racism

Providers shared observations of colleagues’ body language or tone changing in interactions with First Nations patients, as well as approaching the care interaction with negative emotions such as anger. Providers also mentioned staff having a lack of patience with First Nations patients. One nurse described a particular attitude or approach to First Nations patients, saying: “....stereotypes are sort of applied to patients and their family. Maybe a lack of patience or a lack of…it may be subtle but it’s also a barrier to, like we jump to conclusions potentially or we jump to a judgement about this [First Nations] patient and their family.” (N2) Another provider summed up that “as a general rule, our Indigenous patients probably don’t get treated as courteously and as professionally as our non-Indigenous patients.” (P9)

Almost all providers mentioned that the fast-pace of the ED impacts patient experience, and these sentiments were frequently expressed in relation to ideas about First Nations patients’ care expectations or communication styles. One nurse said, “I would say that that’s definitely a challenge in terms of them coming to the emergency room is that we’re not a place where we have a lot of time.” (N2) A physician described the care interaction as one where “I’m trying to get somebody to make a decision so that we can move on to the next step. But they’re trying to get me to hear them so that I like understand what they need as a person. So I think it causes friction.” (P7).

Some providers contextualized that from their perspective the ED care process may not fit First Nations communication and decision-making styles, as they understood them.

“I think it’s a cultural thing, but you know, in Western, I mean in non-First Nations interactions, there’s often a rapid back and forth flow of discussion, or like the question and answer has kind of got a different rhythm than it does in some First Nations patients. And that slow cadence is probably, is probably annoying or a negative feature that further exacerbates the problem. Because I think you need more patience with a ‘ce’ to talk to First Nations patients, because they’re more thoughtful, they think a bit differently, they approach issues differently, they may want to consult with family or friends.” (P11)

Some providers also felt that they may be more open to, or communicate better, with patients who they perceive to be of the same “race” as themselves. One highlighted making judgments about a patient’s capacity based on their way of speaking.

“[A patient] had a, you know, a definite audibly First Nations way of speaking right, … her accent, if you will, was characteristically First Nations. And after quite some time of interacting with her, it became, I became aware she was actually a University professor at [Institution] or something. I was surprised by that.” (P9)

The provider reflected on this as evidence of personal bias (see fuller quotation in Additional file 1: Appendix).

##### Differential medical care

A majority of providers reported that they believed that First Nations patients receive appropriate care most of the time, but many also noted exceptions when racism, stereotyping or leaving without being seen impacted care. Three physicians made the point that if you define appropriate care as clinical care, First Nations patients receive appropriate care, but if social needs are considered, they do not.

“Like I would say that [First Nations patients] get the best care that we’re capable of giving. But the system isn’t really designed to meet the need, their need… The health system is designed really well for patients who have good primary care, who have the resources to be able to go to hospital, get their assessments, get their medications filled, and um see their specialists if they need them in follow up… it is generally clinically appropriate for the problem that presents. But if you take a clinical perspective that you have to take social factors into account, then no.” (P13)

Providers also noted that racism can jeopardize clinical care. A provider described witnessing the following example of overt racism jeopardizing care during their residency:

“I saw a girl who was First Nations who had abdominal pain and I was thoroughly convinced she had appendicitis…. And the physician who I was working with…was running her mouth about “Indians” like going on like a big racist rant. Like out loud at the nurses’ station in the emergency department. And the patient was in a bed that was just kitty corner to the desk and she can hear what was going on and she got up and she took the IV out of her arm and she was bleeding on the floor… And she left. And I said to her, ‘can you please go to another hospital? Like please, I understand why you’re leaving but please go somewhere else, you need to receive care’. And I don’t know what happened to her.” (P7)

The same provider described a case of Kawasaki’s disease being missed in ED visits prior to their seeing the patient, and linked this missed diagnosis to anti-Indigenous racism (see Additional file 1: Appendix). Another provider described cases of not addressing issues of domestic violence (see Additional file 1: Appendix). Other examples of inadequate medical care being provided to First Nations patients were also given, related to less thorough investigations.

“And I would say that we often don’t take the time to try to understand exactly what’s going on with that patient, whether its, you know, they’re intoxicated, whether they’re, for some reason there’s a mental health concern, that sort of thing. We don’t have the time to try to understand that. And I think when race comes into that, we may even make a more quick judgment, unfortunately.”(P15, fuller quote in Additional file 1: Appendix)

Providers spoke particularly frequently about how stereotypes of First Nations persons as substance using impact diagnosis and care practices. One provider gave examples of two First Nations patients who were found unconscious and assumed by paramedics to be suffering from a substance related problem, but were diagnosed in the ED with meningitis and septic shock respectively (see Additional file 1: Appendix). Another provider described that if the perception at triage is that the patient is intoxicated, “the patients will wait a long time in the waiting room. They will park them in a room in a chair for 6, 7, 8 hours, 10 hours sometimes” before seeing a physician. (P6) A second provider said that “… if you have an intoxicated First Nations, its usually not a big deal, they usually disappear. So people just almost ignore them, or if you literally are kind to them and give them a sandwich they are so grateful, they leave.” (P3).

Explicit differences in the way intoxicated First Nations patients are treated, compared to white patients, were also described:

“… white kid with alcohol on board versus a Native kid with alcohol on board. White kid with alcohol on board is belligerent and everything else but they find a way to deal with him. Native kid is slightly belligerent, come in, just hold him down, sew in the sutures and call the RCMP and send him to the drunk tank….And the white kid would go home with his parents.” (N10)

### Power differentials in the ED

Providers described the power of physicians over patients within the ED.

“I think the ED is, for all patients, has, there’s a huge power imbalance…. Patients are like just sick and they want help… and then we take all their clothes off and put them in a gown. And then we like leave them for hours, waiting for tests. And they’re so vulnerable and in such pain. And then I come in and I have like a uniform, and I have a stethoscope around my neck [which is] to me, a symbol of authority… And I ask them, I can ask them anything. I can ask you anything in the emergency department and expect you to answer. I can ask you about your sexuality. I can ask you about your sexual practices. I can ask about your gender identity, I can ask your cultural identity. … Where do they live? Where do they get their money? … I can ask you any question in the ED and I expect you to answer. If you don’t answer my questions in the ED you’re labelled as difficult or uncooperative.” (P7)

#### Provider Power to Advocate for Care or not

Recognition of provider power was also evident in providers’ descriptions of determining how much care to offer first nations patients in the ED, and actions they might take to advocate for first nations patients to receive care. A physician described the case of a patient they recalled as “visibly First Nations” with a trimalleolar fracture as “a good example.” they describe that the patient needed surgery, but that the surgeon set eligibility criteria that the patient would likely be unable to meet due to their social determinants of health (housing and substance use). “I waited until we had a bed available on our hospital care team and asked them to admit the patient and do some social stabilization, get [an addiction team] involved and at least try to give them a chance to have this surgery.” (P13, full quotation in Additional file 1: Appendix).

### Provider Attitudes Towards Care of First Nations Patients

Providers expressed varying degrees of awareness of Canada’s history of colonialism, systemic racism and resulting impacts on First Nations members’ health. A spectrum of provider attitudes towards First Nations care were described, ranging from belief that care is appropriate, through frustration and ambivalence, to motivation to do more for First Nations patients.

One provider noted that there were lesser efforts for First Nations patients due to “ambivalence,” saying “its apathy, it’s a sense of hopelessness, its ummmm, it’s a sort of a frustration with you know, with these things, with efforts not translating into benefit [for First Nations patients].” (P11).

A few providers recognized historical injustice towards First Nations people in Canada but denied personal responsibility for “the past” and expressed that they did not know how their care practice should change.

“Healthcare-wise, I personally….was [pre-school age] by the time residential schools closed. Both my parents are from [outside North America]. So I personally am like, I don’t know how I can make up for things that people have done in the past that I personally didn’t actually have anything to do with. … I understand there’s a lot of talk about well it’s a systemic thing, but it doesn’t actually help me to offer better healthcare or to you know, say ‘okay these are your differences, these are my differences, this is how we can work together.’ I feel like it’s still kinda just over our heads…” (N1)

In contrast, some providers noted taking extra care and time to ensure that First Nations patients were receiving equitable care. “I want to make [First Nations patients’] experience like as best as I possibly can so that they, like, you know if they have had negative experiences in the past that I’m at a minimum not adding to [that].” (P4).

A few providers reflected on their role as healthcare providers in light of the history of colonialism uniquely impacting First Nations patients, as in the following quotation. “Well, you’re the white doctor in a position of power …and you represent a very unfortunate history of, kind of, colonial violence and it’s important to be conscious of that…” (P17).

Another provider reflected on the importance of recognizing that they are not exempt personally from racist thinking in pursuing “cultural safety” in the care they offer.

“… I think , it’s so, so bad to be racist in our culture that we like get really afraid of confronting our biases because we just have to like, act like we’re not racist. Whereas cultural safety just accepts that we’re all racist, we all have these like biases, and it takes some of that pressure away like…. You have this like racist bias that was like implanted into you by like a system that discriminates against Indigenous people.” (P7)

### Provider efforts to address racism and systemic issues in First Nations care

#### Efforts to mitigate past negative experience with healthcare

Our interviews documented examples of activities individual providers take to facilitate care for First Nations patients. In the following quotation, a provider discusses making additional effort in communication.

“you hear you know, ‘oh this patient is refusing their blood work, this patient doesn’t want to stay anymore,’ or whatever it is. And so instead of just saying, ‘well screw it…’ I’ll go back and have another conversation… I’m not going to do that kind of song and dance with every patient. But I will do it with patients where I think that there is a history of poor interactions with the system.” (P14)

In an effort to prevent First Nations patients from feeling stereotyped when asked about substance use, another provider described that:

“….sometimes I’ll say, ‘in addition to the medical questions, I need to ask about other things that could impact patient care, or impact your health, such as smoking and drinking’. So I try to couch it and preface it. And I think, one strategy is you can ask them for permission to ask. I’ve tried that.” (P6)

#### Effort to mitigate systemic barriers to care

Some providers described efforts to address what they saw as systemic barriers to care, such as transportation barriers.

“… sometimes it’s not being able to contact the families, so having a contact number…So, then there’s an inability to contact them to say, ‘is there an issue?’ Because if there’s an issue with getting a ride, we can, you know, we can provide a taxi chit, so that you can get to the hospital so we can get you here so that we can provide care.” (N5)

Further examples are given in the Additional file 1: Appendix. We categorize efforts providers mentioned according to the barrier they address in Table 2**.**

**Table 2:** Barriers to care and provider mitigation efforts

Barriers to Healthcare for First Nations Patients	Examples of Provider Efforts to Mitigate Barriers
Transportation	• Arranging transportation for the patient (e.g. through providing resources or connecting with transportation services).(P4, N5, N8, N10, P11, P13, P14, P15) • Arranging for patients and their families to stay overnight in ED, where appropriate.(N5, N8) • Advocating for specialists to book patients with long travel distances later in the day.(P7) • Arranging inter-facility transport.(P8) • Advocating for a patient to be admitted when follow up is difficult, or to be admitted to a hospital closer to their home, when this is the patient’s preference.(N2, N5, P17)
Communication	• Taking more time or having more conversations with First Nations patients.(N2, P3, P4, P6, P7, P9, N10, P15) • Updating contact information at each visit.(P13, P14) • Calling family members to leave messages if the patient has no phone.(P13) • Building relationships with clinics in First Nations communities. (N5, N8, N10)
Resources	• Writing notes for patients to access coverage for transportation costs.(P7) • Educating themselves on what resources are available for First Nations patients and referring patients to these resources.(P3, P7)
Access to primary care	• Communication about follow up care needs with staff at the First Nation Health Centre (e.g. a Nurse Practitioner), who are often more available and consistent than physicians (who may rotate into and out of communities).(N5, P7) • Arranging for patients to come back to ED for follow up where primary care is not readily accessible.(P7, N10) • Writing longer prescriptions for patients who lack access to primary care.(P7) • Communication with family physician or outpatient clinic to encourage continuity of care.(P15)
Discrimination	• Taking additional time and effort with patients who have had negative experiences with the healthcare system.(N2, P7, N8, P9, P11, P14) • Not relying on past providers’ potentially biased diagnoses.(P7) • Sensitivity in how questions about substance use are framed.(P6, P14) • Challenging negative comments and use of racial slurs by colleagues.(P3, P7)

## Discussion

ED providers described racism and stereotypes they had witnessed, failures to meet First Nations patients’ emergency care needs and systemic barriers that impact emergency care for First Nations patients. EDs were also described as places where First Nations patients are exposed to disrespect through tone and body language, may encounter overt racism (e.g. through racist slurs), are subject to force (e.g. held down while sutures are put in), and (just as harmfully) may be neglected or not taken seriously. Our data also shows that EDs are places where First Nations patients may encounter police and Children’s Services agents who can impact their lives long after the ED encounter.

Although we failed to recruit First Nations providers and ended our data collection sooner than intended, our sample is sufficient to generate new understandings about ED provider perspectives on emergency care for First Nations patients. Smaller sample sizes are warranted when participants are relatively homogenous and the topic relatively focused (as is the case in our study) [52] and a sample of 8 to 20 has been recommended for qualitative studies in emergency medicine [43]. Moreover, our lengthy interviews are particularly rich data sources [53], which may allow a deeper exploration of the topic than would be the case using a larger number of shorter interviews.

Specifics of ED providers’ stereotypes about First Nations patients, and the way these impact care delivery, were a main finding of our research. Our findings align with previous findings that healthcare providers hold stereotypes of First Nations patients as misusing healthcare and as substance using [14, 16–18], while showing that these stereotypes are particularly salient within ED care. It seems to us that these stereotypes interact with emergency providers’ efforts to categorize patients and ration scarce care resources, as documented in sociological studies of EDs [37–40], in ways that disadvantage First Nations patients.

Sociological studies of EDs have shown that practically any patient seeking ED care must strive to present themselves as both a legitimate ED case (i.e. as someone with a health condition which is within scope of emergency medicine) and as personally deserving of care [37–39]. Among other considerations, patients may be expected to demonstrate that they are responsible consumers of limited ED resources, that they want to get well and will cooperate with care instructions [39]. The idea that providers allocate care based on their expectations for patient behavior is implicit when P7 describes their power to question patients, and the risk that unresponsive patients run of being “labelled difficult or uncooperative.”

Our interviewees also described that from their perspective many patients do not understand the purpose of EDs, expect care that the ED cannot provide or desire more time than the provider feels empowered to allocate to them. By expressing these concerns, our interviewees demonstrate that providers’ perspectives on what ED is for and capable of impact how care interactions with particular patients unfold. Such ideas have been shown to vary among providers, and to be shaped by what resources providers have access to in a particular ED at a particular time [38, 39]. As such, the same patient with the same presenting complaint may receive different care on one day compared to another “according to such factors as the doctor [seen], the number queuing in the waiting room, and the interpretation made of his or her social situation.” [38] Our research shows that First Nations patients face immediate additional difficulty in securing ED care compared to other patients, given stereotypes of First Nations members as ‘misusing’ ED. When these stereotypes are applied, routine ED provider practices of categorizing patients and rationing care pre-dispose First Nations patients to receive less or lesser quality emergency care.^6^

Similarly to stereotypes of ED misuse, stereotypes related to substance use disadvantage First Nations patients within EDs. The perspectives shared that intoxicated First Nations patients are often “ignored,” left to wait for extended periods, or expected to “disappear” from the ED are particularly disturbing. They appear to reflect categorization of patients who use substances (or who are perceived as using substances) as less deserving of ED care than others. As a result, their access to care is restricted through strategies of care rationing including inattention, long waits and treating cases where patients leave the ED without receiving care as acceptable. Such practices do not appear to differ from those observed in mid-Western American EDs in the 1970s, when Mannon documented that substance using patients are frequently classified as “management” problems, rather than medical patients, to be moved out of the ED with as little disruption to other ED operations as possible [55].

The rationing of care that can occur when patients are classified as substance using is especially concerning in light of providers reports that medical problems are sometimes misidentified as substance use, and the role assumptions of substance use played in First Nations members deaths outlined earlier.^7^

Separately from findings related to stereotypes and care rationing, we also heard reports of stereotypes about First Nations parenting held by ED providers. Previous studies have reported similar stereotypes about First Nations parents’ supposed inability to care for children [58], and First Nations members' concerns about being accused of abusing or neglecting children when seeking medical care for children [16, 59]. A historical review of Canadian news articles found variations of this stereotype used throughout Canadian history to argue the “inferiority” of Indigenous peoples and justify the mass removal of Indigenous children from their homes [60]. The reported continued presence of this stereotype among ED providers is especially concerning given the high percentage of children in Alberta’s custody who are Indigenous (69% in 2016) [61]. Providers’ descriptions of First Nations women being stereotyped as having many children are also troubling in light of contemporary findings of sterilization of Indigenous women without informed consent in neighboring Saskatchewan [62].

Many of the perspectives and examples shared by providers in our sample call into question the degree to which providers caring for First Nations patients are meeting core competencies required by codes of ethical conduct for physicians [63] and nurses [64]. For example, providing safe and compassionate care, respect for all persons, honoring dignity and commitment to justice, professional integrity and competence, personal inquiry and reflection. We heard that providers often felt apathy and frustration when they perceived that their care is “not translating into benefit” for First Nations patients.

### EDs as a site of continuing colonialism

Provider expressions of apathy regarding First Nations care documented in our work align with Razack’s analysis of inquiries into the deaths of Indigenous persons in police custody [2]. Razack found that settler failures to provide the necessities of life for Indigenous persons in custody were often tied to provider feelings of inability to care for individual Indigenous persons and wider societal discourses that frame Indigenous *peoples* as irreversibly damaged [2].

Razack’s work shows that failure to provide care is as much a part of the way colonialism functions within Canada as, for instance, direct violence enacted against Indigenous peoples. “It goes without saying that a damaged and dying people cannot be entrusted with self-governance and stewardship over land” such that descriptions of “Aboriginal dysfunction” reinforce colonial occupation [2].

As Foucault has described [65], while states have historically relied on the sovereign power to kill (as Canada has when armed military and police have enforced state policies and legislation), state power is currently expressed just as much through exercise of power to “let die” by withholding lifesaving care and (at a policy and government level) withholding resources. The current set up of the healthcare system (including lack of primary care available to most First Nations), and widespread stereotypes of First Nations members, predispose emergency nurses and physicians to contribute to settler colonialism when apathy and frustration set in.

To the extent that EDs are places where First Nations patients face direct violence, or where the process of state removal of children from their families and communities begins, EDs also continue the colonial oppression of First Nations.

### Barriers to change

Our study identified particular barriers to change that can be contextualized through the wider literature. In a study of medical students’ understandings of stereotypes toward First Nations patients, Ly and Crowshoe found that students believed these stereotypes to be rooted in realities [66]. Providers in our sample also appeared to see stereotypes about First Nations patients (particularly around substance use) as grounded in realities, even as many acknowledged how racial stereotypes may result in First Nations patients not receiving appropriate medical care.

Importantly, our findings also suggest that anti-racist education must go beyond simple transfer of facts. While some providers in our sample were able to describe facts about determinants of First Nations health (e.g. naming colonialism, giving dates residential schools were open), this did not necessarily translate into an understanding of how an individual provider might provide better quality and culturally safe care. Indeed, in some cases these understandings were utilized to deflect responsibility from providers, as in the cases of two providers who described not being responsible for residential schools. As such, simplistic deficit based narratives about Indigenous peoples, historical harms and contemporary social determinants of health can contribute to colonialism by providing alibis for poor care.

### Content and strategies for education on anti-Indigenous racism and colonialism in the context of emergency medicine

Ly and Crowshoe suggest that anti-racist education “approaches should be flexible to meet the needs and challenges presented by the contexts in which they are taught.” [66] Relatedly, Berg and colleagues found that ED providers and leaders desire “cultural competency” training to be tailored to the ED context [67]. Our results also mirror findings that healthcare providers desire education to include concrete direction on how their own practice should change [68]. As such, we recommend development of ED specific anti-racism education. We would stress that inclusion of First Nations educators in creating anti-racist training for ED providers is crucial. Such training could include selected cultural components, to give providers locally applicable knowledge of First Nations ways of speaking [69–72] and behavioural norms [73] that may impact shared decision making or diagnostic assessments in ED [74]. However, critical education regarding ongoing systemic racism and colonialism should be prioritized [75]. Moreover, this training must show providers that racism and colonialism interact, through stereotypes, with processes of care rationing and patient categorization that ED providers understand as necessary parts of their practice. This will prevent ideas about the limitations of EDs and the proper use of EDs from becoming routine justifications for offering less, or lesser quality, care for First Nations patients.

It is especially important for providers to know that the explicitly racist *Indian Act* [76] makes Indigenous healthcare the responsibility of the Federal Government, whereas the *Constitution Act 1867* makes healthcare in general a provincial responsibility [77]. First Nations are thus left in a jurisdictional gap, as neither the federal nor provincial governments take responsibility for provision of health services [77]. In 2011, the Auditor General of Canada found a lack of evidence that the Federal Government is committed “to providing services on reserves of the same range and quality” as enjoyed elsewhere [78]. The Auditor General further found that Federal funding mechanisms make First Nations organizations responsible for healthcare without adequately or sustainably resourcing them for success [78]. In 2018, the Auditor General described the lack of progress that had been made in addressing these issues as “beyond unacceptable.” [79] In turn, limited access to primary care for First Nations members drives greater ED use [18].

ED providers need to understand these realities in order to avoid blaming First Nations patients for factors that are beyond their control [66], to challenge stereotypes of “misuse” of ED, and to deliver high quality care to each patient. Beyond the ED, rather than succumb to frustration, patient blaming or apathy about care of First Nations patients, providers could push their organizations (e.g. associations, colleges, unions) to form alliances with First Nations organizations to advocate for systems change. Health systems leaders in turn need to understand policy and political environments so that jurisdictional gaps in care can be highlighted and addressed at subnational and national levels.

In terms of changes that may be made within ED environments, we would point to examples some participants gave of actions they take (Table 2) that could be replicated by other providers. We have found it useful to generalize from the specific practices providers described and to categorize their efforts in terms of Ford-Gilboe and colleagues’ framework of Equity-Oriented Healthcare consisting of Trauma- and Violence-Informed Care (TVIC), Culturally Safe Care, and Contextually Tailored Care (See Table 3) [46]. TVIC works to “acknowledge and address the intersection and cumulative effects of interpersonal… and structural… forms of violence on people’s lives and health.” [46] In the ED context, interpersonal traumatizing events would include discriminatory comments by care providers, while structural violence takes the form of reduced access to primary care [80, 81] and, potentially, the rushed pace of the ED encounter itself. Culturally safe care “moves beyond culturally sensitive approaches to explicitly address inequitable power relations, racism, discrimination, and ongoing effects of historical and current inequities within healthcare encounters” [46]. Finally, contextually tailored care, “expands the individually focused concept of patient-centered care to include offering services tailored to the specific health care organization, the populations served, and the local and wider social contexts” [46]^8^. These three concepts provide a framework through which to conceptualize practices that make care equitable. Using this framework may help providers and departments to innovate and expand upon the equity-oriented practices described by participants in our study.

**Table 3:** General emergency department provider strategies categorized as dimensions of equity-oriented healthcare (Ford-Gilboe Framework)

Dimension of Equity Oriented Care	ED Provider Strategies
Trauma and Violence Informed Care	• Recognizing past negative experiences with the health care system. • Making efforts to address concerns that may lead patients to decline or leave care. • Involving patients in shared decision making.
Culturally Safe Care	• Recognizing that there can be differences in First Nations communications styles and behavioural norms (which will vary between peoples and communities), which fast-paced ED processes are not designed for. • Adjusting care approach to allow more time and ensure shared decision making. • Sensitivity in how questions about substance use are framed. • Advocating with colleagues for better understandings of social determinants of health, including discrimination. • Attention to power imbalances and their impact on the care encounter. • Reflection on personal biases and self-correcting biased assumptions during care interactions.
Contextually Tailored Care	• Ensuring follow up appointments are at times and in places that patients can attend. • Ensuring the ability to communicate with the patient after discharge. • Ensuring continuity of care with next provider. • Providing transportation options. • Providing documentation patients need for other services. • Addressing social determinants of health that may prevent needed follow up care.

Such equity-oriented practices could be incorporated into training of ED care providers and departmental policies, and mandated as part of appropriate care.

### Going beyond responses by individual providers: department and system accountability

As valuable as individual providers’ efforts to deliver equity-oriented care are, we would advocate that they are not enough and that it is unfair to place the responsibility of addressing racism and ongoing colonialism on providers as individuals. Moreover, as Lavoie described in a different context, when providers are made responsible for deciding how far to go in addressing client needs in a situation of unclear accountability and scarce resources this will only increase the arbitrariness of care from patients’ perspectives [83].

We would therefore not advocate for simple consciousness raising among ED providers or for simply increasing the number of providers who make *individual* efforts to address systemic barriers to care. Instead, we argue that systems themselves must also change to address the social determinants of First Nations health, perhaps incorporating the advice of advocates for “social emergency medicine” [84–86]. This may involve consideration of whether the usual “rapid back and forth” speed-driven ED care encounter is itself culturally unsafe for many First Nations patients, or indeed, unsafe for many patients in general. New work is needed to ensure that care rationing and patient categorization practices in EDs are informed by medical assessments and equity-oriented principles, and decoupled from racist ideas. EDs could develop a standardized set of practices expected of each department as a whole (rather than of individual providers) to ensure consistent care for First Nations patients and others. Following the Māori framework for health equity [87], which advocates that improving healthcare equity requires change at practitioner, organization and systems levels, we present recommendations for ED providers, EDs and health systems in Table 4.

**Table 4:** Recommendations for ED Best Practices (Department Level)

**Recommendations for Delivery of Emergency Care for First Nations Patients**	
**For Providers**	
• Treat each patient as a unique individual for purposes of diagnosis and shared decision making.	
• Conduct complete investigations at each ED visit.	
• Learn about and reflect on stereotypes and biases, and self-correct during care encounters.	
• Learn about history of colonialism and contemporary realities of First Nations – including locally.	
• Recognize that:	
- Resources outside ED are not always the same for First Nations patients as non-First Nations patients (e.g., primary care, transport).	
- First Nations patients often have past negative experiences with healthcare.	
- Communications styles and behavioral expectations vary cross-culturally.	
- Stereotypes can be activated by your words and actions regardless of your intent.	
• Modify approach and care plan accordingly.	
• Be very cautious about reporting a family to Children’s Services or calling police, given stereotyping and adverse consequences in child welfare and criminal justice systems.	
**For Departments**	
• Take for granted that racism impacts health and healthcare.	
• Work to build formal relationships with First Nations communities. These could serve to:	
- familiarize providers with the realities that First Nations patients face and the resources that are available to them.	
- enable the ED to understand and work to address the expectations of the community/community health services for emergency care.	
- facilitate inclusion of First Nations members in department committees and governance.	
• Ensure providers have resources necessary to offer equity-oriented care for all patients and presenting complaints.	
• Develop training on local resources, services and funding sources available for First Nations patients.	
• Create standard discharge pathways for First Nations patients that involve:	
- sensitively enquiring whether the patients’ living environment is suitable to healing, and involving social supports where it is not,	
- considering patient access to transport home following the ED visit, and providing support where needed (e.g. assistance calling friends/family for transport, a safe place to wait until transport arrives, taxi vouchers)	
- considering patient access to transportation when developing plans for follow-up and ongoing care, and providing related supports (e.g. asking specialists to schedule follow up at times and places patients feel they can attend, accessing health system resources like inter-facility transport),	
- follow up communication with the next provider,	
- follow up communication with Health Clinics in First Nations communities,	
- enquiring whether patients require physician letters (e.g., for time away from work, medical reimbursement or other services),	
- involving Health System Navigators or Indigenous Health Liaisons in discharge and follow up planning.	
• Advocate within the healthcare system for resource allocation and quality improvement efforts for First Nations care - within and outside ED.	
**For Emergency Care Systems**	
• Create safe and well-moderated ED specific training to help providers identify prevalent stereotypes of First Nations patients and develop anti-racism skills. This training should involve First Nations educators and professionals, and address specific problematic ideas:	
- that colonialism happened only in the past.	
- that history is not relevant to the present.	
- that stereotypes about First Nations peoples are rooted in reality.	
- that encounters with particular patients can be used to draw conclusions about the social group the patient is perceived to belong to.	
- that generalizations about social groups can be applied in the diagnoses and medical treatment of particular patients.	
• Promote education on racism and colonialism, and not only First Nations languages, English dialects, communication styles and behavioural norms.	
• Create standard forms, planning documents, information resources and other tools to facilitate the above.	
• Provide departments with resources necessary to offer contextually tailored care and address patient barriers to continuity of care (e.g., transport and other resources).	
• Ensure appropriate processes for reporting and restorative follow up of racist behaviour that ensure anonymity of the reporting party.	
• Recognize First Nations’ sovereignty by ensuring that First Nations governments are robustly involved in decision making about the resources, goals and form of the emergency care system in keeping with the United Nations Declarations of the Rights of Indigenous Peoples.	

Steps at a systems level could include making accreditation of educational and healthcare institutions contingent on having detailed anti-racism and anti-colonialism plans in place, and on measurable efforts to address racism (including tracking numbers of complaints of racism and their resolution). Furthermore, First Nations must have a role in governing emergency care services in keeping with Joyce’s Principle, as described in the introduction, and the United Nations Declaration of the Rights of Indigenous Peoples (UNDRIP). UNDRIP Article 23 states in part that “[I]ndigenous peoples have the right to be actively involved in developing and determining health… programmes affecting them...” [88]. This could be realized in part through the creation of a First Nations Health Ombudsman, or other institutional mechanisms for First Nations oversight of the health system. Moreover, systems approaches to improving First Nations healthcare will require reallocation of resources, including outside EDs, and especially for First Nations access to appropriate and culturally safe primary care.

Future health system work could additionally involve efforts to create well-funded First Nations run emergency care settings, on and off-reserve, guided by First Nations principles and values. A recent analysis of Indigenous-led healthcare partnerships in Canada [89] provides examples and recommendations for cultivating and expanding Indigenous-led models of care.

### Limitations

Participation in this study was voluntary and providers knew the study addressed racism. As such, we believe that our sample mainly includes persons who feel that racism is a problem in emergency care. We cannot assume that the level of understanding of racism, residential schools and barriers to healthcare expressed by our sample can be generalized to all ED providers. No providers identified as First Nations, and we believe that distinct findings would arise from interviews with First Nations ED providers. As our study is partnered with First Nations organizations, we have not specifically explored provider perspectives on care of Métis and Inuit patients. Providers did not always appear to be conscious of distinctions among Indigenous peoples, and often seemed to be speaking of Indigenous patients in general during interviews, raising questions about the specificity of our findings to First Nations emergency care.

## Conclusions

Our findings demonstrate that gaps in understanding and negative attitudes toward First Nations members exist even among providers who are motivated to pursue health equity. We demonstrate that racist stereotypes of First Nations members interact with ED provider practices of categorizing patients and rationing care to put First Nations patients at risk of poor care, and thereby reinforce settler colonialism. Our results can aid development of anti-racist and anti-colonial continuing professional education and standardized equity-oriented care practices specific to EDs. These practices would go beyond necessary education on residential schools, intergenerational trauma, colonialism and systemic racism to provide concrete tools and content specific to the ED environment. Beyond the ED, systems responses to barriers to First Nations healthcare are required, including First Nations-leadership within health services.

## Supplementary Information

**Additional file 1.**

**Additional file 2.**

## Data Availability

The data that support the findings of this study are available from the Alberta First Nations Information Governance Centre but restrictions apply to the availability of these data, which were used under license for the current study, and so are not publicly available. Data are however available from the authors upon reasonable request and with permission of the Alberta First Nations Information Governance Centre.

## Abbreviations

ED: Emergency Department
N: Nurse
P: Physician
AFNIGC: Alberta First Nations Information Governance Centre

## Footnotes

1 A recent class action lawsuit pursuing compensation for the damages the hospitals caused names seven such hospitals within Alberta, where our study was conducted [9].

2 First Nations team members have noted that this definition of racism comes from Western social sciences, and that because racism is discussed from Indigenous perspectives by Indigenous peoples and using Indigenous languages, there will be alternate Indigenous definitions of racism. For instance, Blackfoot and Cree language definitions. We do not develop such definitions here, but recognize the importance of flagging our use of a Western definition in this paper, and the degree to which this shapes our findings.

3 AFNIGC provides this disclaimer: “Parts of this publication are based on data and information from the ‘Understanding and Defining Quality of Care in the Emergency Department with First Nations Members in Alberta project. The analyses, conclusions, opinions and statements expressed herein, however, do not necessarily reflect the views of AFNIGC and are solely those of the author(s). Statistics reproduced from this document must be accompanied by a citation of this document, including a reference to the page on which the statistic in question appears.”

4 One interview conducted with an Indigenous health liaison is not included in this sample, and will be analyzed in another product alongside First Nations health director and community member perspectives. Thus our interviews are numbered to 17, although we have 16 participants.

5 As stereotypes of First Nations members as substance using may contribute to over-diagnosis of substance use in ED visits by First Nations members, it is difficult to know how accurately such diagnoses reflect true proportions of substance use among First Nations members’ ED visits.

6 As a research team, we would further argue that the concept of ED misuse is in itself problematic, as patients define their need for ED care based on their circumstances, knowledge and perceived care options. As the Royal College of Physicians and Surgeon’s of Canada states “Emergency Medicine is a cornerstone of Canadian healthcare, providing universal access to care for all patient presentations, including underserved and/or disadvantaged populations, at all times.” [54]

7 During the course of our data analysis, in early 2021, Lillian Vanesse died in a rural Alberta hospital. The Aboriginal Peoples Television Network reports that after she arrived with lung related symptoms, staff focused on her methadone prescription [56]. At the time of this writing Alberta’s physician and nurse colleges are each separately investigating [57].

8 Notably, in more recent work the originators of the equity-oriented care framework have argued that harm reduction approaches that de-stigmatize substance use are an additional key dimension of equity-oriented care [82]. Harm reduction strategies were not explicitly described by providers we interviewed. However, we regard developing strategies for de-stigmatizing substance use and serving substance using patients within ED settings as an important area for future work. This is especially true given the tremendous role stereotypes of substance use play in discrimination against First Nations patients.

## References

[1] Lawrence B, Dua E. Decolonizing antiracism. Social Justice. 2005;32(4):120–43.

[2] Razack S (2011). Timely deaths: medicalizing the deaths of Aboriginal people in police custody. Law Culture Humanities.

[3] Truth and Reconciliation Commission of Canada (2015). Honouring the truth, reconciling for the future: summary of the final report of the truth and reconciliation commission of Canada.

[4] Canadian Institutes of Health Research, Natural Sciences and Engineering Research Council of Canada, and Social Sciences and Humanities Research Council of Canada. Tri-Council policy statement: ethical conduct for research involving humans, chapter 9: research involving the First Nations, Inuit and Métis peoples of Canada. 2018.

[5] Lux MK. Separate beds: a history of Indian hospitals in Canada, 1920s–1980s. Toronto: University of Toronto Press; 2016.

[6] Daschuk J. Clearing the plains: disease, politics of starvation, and the loss of Aboriginal life. Regina: University of Regina Press; 2013.

[7] National Inquiry into Murdered and Missing Indigenous Women and Girls. Reclaiming Power and Place: The Final Report of the National Inquiry into Missing and Murdered Indigenous Women and Girls. Canada; 2019.

[8] Granzow K (2021). Against settler colonial iatrogenesis: Inuit resistance to treatment in Indian hospitals in Canada. Anthropol Med.

[9] Lauren P. $1.1B class-action lawswuit filed on behalf of former 'Indian hospital' patients. CBC News. 2018. https://www.cbc.ca/news/canada/toronto/indian-hospital-class-action-1.4508659. Accessed 9 June 2022.

[10] Czyzewski K. Colonialism as a broader social determinant of health. Int Indigenous Policy J. 2011;2(1).

[11] Tjepkema M, Bushnik T, Bougie E. Life expectancy of First Nations, Métis and Inuit household populations in Canada. Statistics Canada. Ottawa; 2019. https://www150.statcan.gc.ca/n1/pub/82-003-x/2019012/article/00001-eng.htm. Accessed 9 June 2022. 10.25318/82-003-x201901200001-eng 31851367

[12] Cameron BL, Carmargo Plazas Mdel P, Salas AS, Bourque Bearskin RL, Hungler K (2014). Understanding inequalities in access to health care services for Aboriginal people: a call for nursing action. Adv Nurs Sci.

[13] Allan B, Smylie J (2015). First peoples, second class treatment: the role of racism in the health and well-being of Indigenous peoples in Canada.

[14] Browne AJ, Smye VL, Rodney P, Tang SY, Mussell B, O'Neil J (2011). Access to primary care from the perspective of Aboriginal patients at an urban emergency department. Qual Health Res.

[15] Goodman A, Fleming K, Markwick N, Morrison T, Lagimodiere L, Kerr T (2017). "They treated me like crap and I know it was because I was native": the healthcare experiences of Aboriginal peoples living in Vancouver's inner city. Soc Sci Med.

[16] McLane P, Bill L, Barnabe C (2021). First Nations members’ emergency department experiences in Alberta: a qualitative study. Can J Emerg Med.

[17] Wylie L, McConkey S (2019). Insiders' insight: discrimination against Indigenous peoples through the eyes of health care professionals. J Racial Ethn Health Disparities.

[18] Turpel-Lafond M. In Plain Sight: Addressing Indigenous-specific Racism and Discrimination in B.C. Health Care. British Columbia; 2020. https://engage.gov.bc.ca/app/uploads/sites/613/2020/11/In-Plain-Sight-Summary-Report.pdf. Accessed 9 June 2022.

[19] Royal Commission on Aboriginal Peoples. Report of the Royal Commission on Aboriginal Peoples. Ottawa: Canada; 1996.

[20] Inquest Report in the Matter of Brian Lloyd Sinclair, Deceased. Provincial Court of Manitoba; 2014. https://www.manitobacourts.mb.ca/site/assets/files/1051/brian_sinclair_inquest_-_dec_14.pdf. Accessed 9 June 2022.

[21] Richardson L, Fennario T. Feds call for inquest into death of Atikamekw mother Joyce Echaquan: APTN online news article; 2020. https://www.aptnnews.ca/national-news/feds-call-for-inquest-into-death-of-atikamekw-mother-joyce-echaquan/. Accessed 9 June 2022.

[22] Richardson L. Would Joyce Echaquan still be alive if she were white? Quebec coroner says ‘I think so’: APTN online news article; 2021. https://www.aptnnews.ca/national-news/would-joyce-echaquan-still-be-alive-if-she-were-white-quebec-coroner-says-i-think-so/. Accessed 9 June 2022.

[23] Council of the Atikamekw of Manawan and the council de la nation Atikamekw. Joyce's Principle 2020. https://principedejoyce.com/sn_uploads/principe/Joyce_s_Principle_brief___Eng.pdf. Accessed 9 June 2022.

[24] Jones C. Confronting Institutionalized Racism. Phylon. 2002;50(1/2):7–22.

[25] National Collaborating Centre for Determinants of Health (2017). Let's talk: racism and health equity.

[26] Griffith D, Mason M, Yonas M, Eng E, Jeffries V, Plihcik S (2007). Dismantling institutional racism: theory and action. Am J Community Psychol.

[27] Feagin J, Bennefield Z (2014). Systemic racism and US health care. Soc Sci Med.

[28] Taiaiake AG. Colonialism and state dependency. Int J Indigenous Health. 2009;5(2):42–60.

[29] Pickering M, Ritzer G (2016). Stereotyping and stereotypes. The Blackwell encyclopedia of sociology [online version].

[30] McLane P, Barnabe C, Holroyd B, Colquhoun A, Bill L, Fitzpatrick K, et al. First Nations emergency care in Alberta: descriptive results of a retrospective cohort study. BMC Health Serv Res. 2021;21(423). 10.1186/s12913-021-06415-2 PMC8096356 33947385

[31] McLane P, Barnabe C, Mackey L, Bill L, Rittenbach K, Holroyd BR, et al. First Nations status and emergency department triage scores in Alberta: a retrospective cohort study. CMAJ. 2022;194(2):E37–45. 10.1503/cmaj.210779 PMC8900783 35039386

[32] Indigenous Physicians Association of Canada and Royal College of Physicians and Surgeons of Canada. First Nations, Inuit, Métis Health Core Competencies: A Curriculum Framework for Continuing Medical Education. 2009. https://www.hhr-rhs.ca/en/?option=com_mtree&task=att_download&link_id=10851&cf_id=68. Accessed 9 June 2022.

[33] Royal College of Physicians and Surgeons of Canada. Indigenous health values and principles statement, 2nd Edition. 2019. https://www.royalcollege.ca/rcsite/health-policy/indigenous-health-e. Accessed 9 June 2022.

[34] Canadian Nurses Association. Aboriginal health nursing and Aboriginal health: charting policy direction for nursing in Canada. Ottawa; 2014. https://hl-prod-ca-oc-download.s3-ca-central-1.amazonaws.com/CNA/2f975e7e-4a40-45ca-863c-5ebf0a138d5e/UploadedImages/documents/Aboriginal_Health_Nursing_and_Aboriginal_Health_Charting_Policy_Direction_for_Nursing_in_Canada.pdf. Accessed 9 June 2022.

[35] Guerra O, Kurtz D (2017). Building collaboration: a scoping review of cultural competency and safety education and training for healthcare students and professionals in Canada. Teach Learn Med.

[36] Richardson LD, Irvin CB, Tamayo-Sarver JH. Racial and ethnic disparities in the clinical practice of emergency medicine. Acad Emerg Med. 2003;10(11):1184–8. 10.1111/j.1553-2712.2003.tb00601.x 14597493

[37] Buchbinder M (2017). Keeping out and getting in: reframing emergency department gatekeeping as structural competence. Sociol Health Ill..

[38] Vassy C (2001). Categorisation and micro-rationing: access to care in a French emergency department. Sociol Health Ill..

[39] Hillman A (2014). 'Why must I wait?' the performance of legitimacy in a hospital emergency department. Sociol Health Ill.

[40] Roth JA (1972). Some contingencies of the moral evaluation and control of clientele: the case of the hospital emergency service. AJS..

[41] First Nations Information Governance Centre. First Nations Principles of OCAP. 2020. http://www.FNIGC.ca/OCAP. Accessed 9 June 2022.

[42] Schnarch B. Ownership, control, access, and possession (OCAP) or self-determination applied to research: a critical analysis of contemporary First Nations research and some options for First Nations communities. Ottawa: National Aboriginal Health Organization; 2005;1(1):80–95.

[43] Cooper S, Endacott R (2007). Generic qualitative research: a design for qualitative research in emergency care?. Emerg Med J.

[44] Vaismoradi M, Turunen H, Bondas T (2013). Content analysis and thematic analysis: implications for conducting a qualitative descriptive study. Nurs Health Sci.

[45] Browne AJ, Varcoe C, Lavoie J, Smye V, Wong ST, Krause M, et al. Enhancing health care equity with Indigenous populations: evidence-based strategies from an ethnographic study. BMC Health Serv Res. 2016;16(1):544. 10.1186/s12913-016-1707-9 PMC5050637 27716261

[46] Ford-Gilboe M, Wathen CN, Varcoe C, Herbert C, Jackson BE, Lavoie JG, et al. How equity-oriented health care affects health: key mechanisms and implications for primary health care practice and policy. Milbank Q. 2018;96(4):635. 10.1111/1468-0009.12349 PMC6287068 30350420

[47] QSR International Pty Ltd. NVivo qualitative data analysis software. Version. 2015;11.

[48] Wilson S. What is an Indigenous research methodology? Can J Native Educ. 2001;25(2):175.

[49] Kovach M. Conversational method in Indigenous research. First Peoples Child Fam Review. 2010;5(1):40–8.

[50] Denzin NK (2010). Moments, mixed methods, and paradigm dialogs. Qual Inq.

[51] Shenton AK (2004). Strategies for ensuring trustworthiness in qualitative research projects. EducInf..

[52] Guest G, Bunce A, Johnson L. How many interviews are enough? an experiment with data saturation and variability. Field Methods. 2006;18(1):59–82.

[53] Geertz C (1973). The interpretation of cultures: selected essays.

[54] Royal College of Physicians and Surgeons of Canada. Emergency medicine competencies. Royal College of Physicians and Surgeons of Canada; 2017.

[55] Mannon JM (1976). Defining and treating "problem patients" in a hospital emergency room. Med Care.

[56] Pimentel T. Investigation underway into how Ojibway woman died while in care of Alberta hospital: APTN online news article; 2021. https://www.aptnnews.ca/national-news/investigation-underway-into-how-ojibway-woman-died-while-in-care-of-alberta-hospital/. Accessed 9 June 2022.

[57] Ritchie J. Alberta man still searching for answers in the death of his wife at the Hanna hospital. CityNews. 2021. https://edmonton.citynews.ca/2021/12/23/alberta-hanna-hospital-death/. Accessed 9 June 2022.

[58] Smylie J, Phillips-Beck W (2019). Truth, respect and recognition: addressing barriers to indigenous maternity care. Can Med Assoc J.

[59] Kurtz D, Nyberg J, Van Den Tillaart S, Mills B (2013). Silencing of voice: an act of structural violence urban Aboriginal women speak out about their experiences with health care. Int J Indigenous Health.

[60] Pfilger A. The framing of Indigenous Canadian families: a historical discourse analysis. Can J Fam Youth. 2020;12(2):69–83.

[61] Office of the Child and Youth Advocate Alberta. Voices for change: Aboriginal child welfare in Alberta: a special report. Alberta; 2016.

[62] Boyer Y, Bartlett J. External review: tubal ligation in the Saskatoon health region. The Lived Experience of Aboriginal Women. 2017. https://www.saskatoonhealthregion.ca/DocumentsInternal/Tubal_Ligation_intheSaskatoonHealthRegion_the_Lived_Experience_of_Aboriginal_Women_BoyerandBartlett_July_22_2017.pdf. Accessed 9 June 2022.

[63] Canadian Medical Association. CMA Code of Ethics and Professionalism. 2018. https://www.cma.ca/cma-code-ethics-and-professionalism. Accessed 9 June 2022.

[64] Canadian Nurses Association. Code of Ethics for Registered Nurses. 2017.

[65] Foucault M (2003). Society must be defended: lectures at the college de France, 1975–76.

[66] Ly A, Crowshoe L (2015). 'Stereotypes are reality': addressing stereotyping in Canadian Aboriginal medical education. Med Educ.

[67] Berg K, McLane P, Eshkakogan N, Mantha J, Lee T, Crowshoe C, et al. Perspectives on Indigenous cultural competency and safety in Canadian hospital emergency departments: a scoping review. Int Emerg Nurs. 2019;43:133–40. 10.1016/j.ienj.2019.01.004 30733006

[68] Diffey L, Mignone J (2017). Implementing anti-racist pedagogy in health professional education: a realist review. Health Educ Care.

[69] Peltier S (2010). Facilitating language and literacy learning for students with Aboriginal english dialects. Cdn J Native Edu.

[70] Ball J, Bernhardt B (2008). First nations English dialects in Canada: implications for speech-language pathology. Clin Linguistics Phonetics.

[71] Fadden L, LaFrance J. Advancing Aboriginal english. Cdn. J Native Edu. 2010;32:143–55.

[72] Fadden L. Communicating effectively with Indigenous clients. Toronto: Aboriginal Legal Services; undated. https://aboriginallegal.ca/downloads/communicating-with-indigenous-clients.pdf. Accessed 9 June 2022.

[73] Wark JNR, Brownlee K (2019). Interpreting a cultural value: an examination of the indigenous concept of non-interference in North America. Int Soc Work.

[74] Brant C (1990). Native ethics and behaviour. Can J Psychiatr.

[75] Curtis E, Jones R, Tipene-Leach D, Walker C, Loring B, Paine SJ (2019). Why cultural safety rather than cultural competency is required to achieve health equity: a literature review and recommended definition. Int J Equity Health.

[76] Indian Act, RSC 1985, c I–5.

[77] Lavoie J, Forget E, Browne A. Caught at the crossroad: First Nations, health care, and the legacy of the Indian Act. J Indigenous Wellbeing. 2010;8(1):83–100.

[78] Auditor General of Canada. Chapter 4: programs for First Nations on reserves. A status report of the Auditor General of Canada to the House of Commons. Ottawa: Office of the Auditor General of Canada; 2011.

[79] Auditor General of Canada. Opening statement to the Senate Standing Committee on Aboriginal Peoples. Study on the new relationship between Canada and First Nations, Inuit and Métis peoples. Ottawa: Office of the Auditor General of Canada; 2018.

[80] Lavoie J, Wong ST, Ibrahim N, O'Neil J, Green M, Ward A. Underutilized and undertheorized: the use of hospitalization for ambulatory care sensitive conditions for assessing the extent to which primary healthcare services are meeting needs in British Columbia First Nation communities. BMC Health Serv Res. 2019;19(1):50. 10.1186/s12913-018-3850-y PMC6339420 30658626

[81] Davy C, Harfield S, Mcarthur A, Munn Z, Brown A (2016). Access to primary health care services for indigenous peoples: a framework synthesis. Int J Equity Health.

[82] Browne AJ, Varcoe C, Ford-Gilboe M, Nadine Wathen C, Smye V, Jackson BE (2018). Disruption as opportunity: impacts of an organizational health equity intervention in primary care clinics. Int J Equity Health.

[83] Lavoie J, Kaufert J, Browne AJ, Mah S, O'Neil J, Sinclair S, et al. Negotiating barriers, navigating the maze: First Nation peoples' experience of medical relocation. Can Public Admin. 2015;58(2):295–314.

[84] Anderson ES, Hsieh D, Alter HJ. Social emergency medicine: embracing the dual role of the emergency department in acute are and population health. Ann Emerg Med. 2016;68(1):21–5. 10.1016/j.annemergmed.2016.01.005 26921967

[85] Hsieh D (2019). Achieving the quadruple aim: treating patients as people by screening for and addressing the social determinants of health. Ann Emerg Med.

[86] Tam V, Targonsky E (2020). Social emergency medicine: a way forward for training. Cjem..

[87] Ministry of Health. Equity of health care for Māori: a framework. Wellington: Government of New Zealand; 2014.

[88] UN General Assembly. United Nations declaration on the rights of Indigenous peoples: resolution / adopted by the General Assembly, 2 October 2007, A/RES/61/295. https://www.un.org/development/desa/indigenouspeoples/wp-content/uploads/sites/19/2018/11/UNDRIP_E_web.pdf. Accessed 9 June 2022.

[89] Allen L, Hatala A, Ijaz S, Courchene ED, Bushie EB (2020). Indigenous-led health care partnerships in Canada. Can Med Assoc J.

