# Supplementary material for: Impacts of racism on First Nations patients' emergency care: results of a thematic analysis of healthcare provider interviews in Alberta, Canada
Source: BMC Health Serv Res. 2022 Jun 21;22:804. doi: 10.1186/s12913-022-08129-5 (PMC9210059; doi:10.1186/s12913-022-08129-5)
Supplement: Supplementary file 1 — Additional file 1. [file 12913_2022_8129_MOESM1_ESM.pdf]

Qualitative Data Appendix: Further Illustrative Quotations

**Racism in EDs**	
ED as a "hotbed" of Racial discrimination	"I would definitely see a bias against the First Nations… I don't know if I can give you specific examples but… you have this [terminology of] "drunk Indian"… "What's in Bed 6 ? It's a drunk Indian, you don't need to go in." (P3).<br><br>"I think that for many, many healthcare providers, right, their interactions with First Nations patients or Indigenous patients, might be colored by stereotypes, racist notions, colonial mindsets and all that sort of stuff" (P9).<br><br>"First Nations patients, ummm, often come to the emergency department with a degree of weariness based on past experience, with almost an expectation that they may not be treated fairly, or be the victims of stigma. And those things are true, and are not true for the non-Indigenous patients." (P11)
Specific Stereotypes held by ED Providers about First Nations patients	<u>Assumption of avoidable or inappropriate ED presentations</u><br><br>"I think I've seen First Nations patients get brushed off as having something not severe. Because….there was this impression that they, like, misused the ED. So if they were coming with their kid, like there's probably nothing wrong with the kid, because like 'here they are again, using the ED.' Maybe not getting as deeply with the history and physical exams because there was, like, already this bias like, nothing was wrong and you guys just run to the ED whenever you need to."(P7)<br><br>"Because there's no transport, [First Nations] people come in by [Emergency Medical Services] but for things again that really aren't, you know, necessarily appropriate to be brought into the ED for." (N1)<br><br>"There's probably demands on the ED, so between you know, this person is not medically sick and does not need to be in the ED, to I've got other things to do… I'm sure there's a combination of everything. There's other patients to take care of as well. This person is taking up a lot of my time. So I'm sure there's a lot of, it's a mixture, I'm sure."(P6)

	"I'm guilty of it too. If someone comes in and is like, I just want to get tested for STIs, or I just want a pregnancy test, I'm like *really*? Like, and you know its easy for me to sit there and be like, are you kidding me? We have 4 chest pains and a lady with a stroke in the back and all that kind of stuff. I mean, that's how we see it, but its not how they see it. So its not fair, right, for us to be that way, but sometimes…we don't really have a choice."(N8)
	Stereotypes related to substance use<br>"This is just something that stands out to me, like we have a lot of patients that come in with ETOH abuse, which means intoxicated. And they come in to kind of sober up or whatever, or they're brought in by ambulance to sober up. And a lot of our patients are First Nations right? So I feel like it, its one thing that is, its very serious, but it seems to be the norm that [First Nations] people come in for that. And maybe its not, people aren't treated as well as they can."(N8)<br><br>"So I've heard that connotation and there is an undertone there that because [First Nations people] have an addiction health issue, addiction and mental health issues, that they're a drain to the system." (P16)
	Stereotypes of First Nations as "bad parents"<br><br>"But [a case of a First Nations parent suffering substance poisoning] sort of reinforced this sort of stereotype that these parents don't have the capacity to care for their children, and of course, drug use, and like, 'well this is just how it goes with these families.' That's sort of the underlying attitude that can be present. Not with everyone but there was a couple of people who were like, 'well of course. What else could you expect?'" (N2)<br><br>"[Sometimes providers say] 'Oh well you know, those parents won't be back because they're going to be out drinking all day' just stuff like that that was very I feel, common place there, was kind of the basic assumption that these people don't care about their kids."(P4)<br><br>"So if you've already been brought up in [town name] and you've been brought up racist towards the First Nations people, then its only going to just impact your racism even more. 'Oh see, like you know, my parents are right. These people don't take care of their children properly. Look at what happened today.'"(N10)

	<u>Differential Medical Care</u><br>"I bet you, if you did the study, ummm First Nations patients spend longer in the corridor with ambulances umm, than non-First Nations. So there's some systematic racism occurring at that level. And you could follow that all the way through. There's probably suboptimal pain management, there's suboptimal education, the threshold for admitting someone to the hospital is probably higher for First Nations and that…we, they probably experience more difficulty getting admitted because there's a concern that it'll be a difficult admission for a variety of reasons, so… yeah, those things are rampant."(P11)<br><br>"[I]f we do get [a First Nations patient] who's the victim of domestic violence, ah, you know they're, its compounded by other kinds of stigma… the healthcare providers, whether it be the nurses or the physicians, often will shrug their shoulder, … and sort of give the impression, well that's just what these people do, sort of thing." (P11)<br><br>"I'm thinking of a kid that I saw who had been seen a few times in the emergency department and had been told that they were having a vaccine reaction. And it's like, over several weeks, and ultimately ended up having Kawasaki's disease. With a coronary artery aneurysm. Just because, I think, when I reviewed the notes, it looked like they hadn't been listened to…. The person who had seen them previously had made like anti-Indigenous comments to me on other occasions and then I saw this kid with a missed diagnosis."(P07)<br><br>"… medical problems being attributed to like alcohol or drugs is something that I've seen or heard. Where people, somebody comes in with an altered level of consciousness, just assume that it's a substance problem not necessarily assume that it could be a medical problem." (P07)<br><br>"I think there probably is a higher chance to assume that First Nations patients are suffering from a drug overdose or intoxication rather than a real medical illness. And I can think of a two specific cases where that was an issue…, both with First Nations patients, where they were initially treated as a drug overdose whereas both patients actually had life-threatening medical illnesses [of meningitis and septic shock] that we were, like it became very clear, *very* early in their emergency department stay, but the story given to us [by paramedics] was much more suggestive of a drug intoxication overdose" (P17)

	"So we do see a lot of people who have, who are Indigenous and have a big drinking problem, right? And so, when you look at someone and say, my clinical judgement is that this person is probably just really drunk, you know, is part of that, like it's hard to pin where that comes from and to what extent that might be underpinned by racist stereotypes. Because if you've got a record on that person and it says that they've been there 12 times in the last 18 days, really drunk, I'm not sure it really matters whether they're Indigenous or not, right? You're making that judgement based on that person's track record." (P09)<br><br>"I think especially in the emergency department there's a lot of pressure around time and resource management. And it creates stress and in stressful situations people often react kind of, react before thinking, I guess is a good way to put it. And an example may be if there's a patient who's agitated and has a history of being here and they may be immediately seen by security and sedated and put into a secure holding room. And I would say that we often don't take the time to try to understand exactly what's going on with that patient, whether its, you know, they're intoxicated, whether they're, for some reason there's a mental health concern, that sort of thing. We don't have the time to try to understand that. And I think when race comes into that, we may even make a more quick judgment, unfortunately." (P15)<br><br>"[I]f that person had a previous visits for, that could be linked to substance use disorder, alcohol use disorder, then they're more likely to be treated more cautiously, for example, for pain control. If there have been previous similar visits they're less likely to get investigated. I mean that's probably all true for non-First Nations as well, its just probably somewhat even more enhanced for First Nations." (P11)<br><br>"If we had a white university student you know, from a middle income background presenting with alcohol intoxication, umm, we'd go to the trouble of you know, contacting the family, making sure the parents were notified, got to… were at the bedside to go over what happened and explain the risk of ethanol abuse and try to make an impact on that young student and their family insofar as the harms of alcohol. If, however, you take the same story and attach it to a First Nations person who is brought in under the same circumstances related to alcohol intoxication, we're probably more, unfortunately they're more likely just to be put at the door, once they're sober. And you know, put a, they're gonna get a label of well this is probably going to happen again, we can't change this trajectory, its not worth the effort. We'll just put them at the door." (P11)

	"[When we would have an intoxicated Firs Nations patent] it was more, let's get him a sandwich, he'll shut up, let's get him out of here because we've got people to see. … I don't know how many care plans we had for First Nations people, probably very few. But it was like, you're there again, sober him up, once they could walk give him a sandwich and get him out of here. Which is not really a good long-term solution to that stuff." (P3)
	Social determinants of health are not addressed<br>"Now if someone from [a First Nation] is having a severe, previously healthy, having severe chest pain, and they're having a heart attack, they're gonna get the exact same care. Except for the fact that they probably have longer transport time. They're gonna be treated the same, exactly the same, as anyone else. But its in that category of violence, trauma, substance or mental health, all of those things that are influenced by social determinants of health, that First Nations patients are getting a raw deal."(P11)<br><br>"I tried to think about this a lot like before the interview, and I think that First Nations patients, when I've felt that appropriate care has been lacking, its largely been due to the social circumstances, whether that's inability to access care because of like addictions issues, or housing issues. So when I thought about this, its more related to their socioeconomic ability to access care, rather than their First Nations identity. If that makes sense."(P17)
Racism Impacting Care	<u>Interpersonal racism</u><br><br>"I would say, maybe it's subtle, and maybe again it's sort of across the board in terms of cultural differences. I think when somebody is, you know, the same race, same language, obviously higher capacity, you kind of, I would say that maybe there is more openness to listening or giving the time." (N2)<br><br>"I know that, like, white people know the words to say to me to like perk my ears, you know what I mean? Maybe they know how to say, like, 10 out of 10 chest pain. Maybe they know how to say fever, or lethargic, right? Where like, maybe, I don't know, this is just a question, maybe people in other cultural groups don't know the right words to say, quote "right" words to say, and so we don't hear them the same way. I've often wondered about that because I think

	sometimes I catch myself taking people less seriously who aren't of a white background, and I'm like, 'oooh, why am I doing that?' And I'm not sure yet." (P7)<br><br>"the challenge with shared decision making is that you have to target it to the level, the patient's level of health literacy…. And so that may be an area where we drop the ball based on cultural stereotypes, right? … I had a patient who, a lovely, older First Nations woman, who, you know, … had a, you know, a definite audibly First Nations way of speaking right, … her accent, if you will, was characteristically First Nations. And after quite some time of interacting with her, it became, I became aware she was actually a University professor at [Institution] or something. I was surprised by that. And I think that's a full-on marker of your unconscious bias, right? I don't think that if that patient had been white, I would have been as surprised to find out that she was a University professor." (P9).<br><br>"I don't know if anyone does it in front of [First Nations patients], but there's the eye roll, there's tone of voice, body language, there's a…disconnect there with what they're supposed to do, what's care…So for example, I think the care is still delivered but maybe not in the manner it can be. Especially…outside of the room…Yeah, outside of earshot or what they think is earshot, but the patients actually hear a lot. Especially… where everything is a curtain, there's very few walls, right, people hear everything. I think people, staff forget that." (P6)<br><br>"I think a lot of us are a little bit bitter because there's just kind of been some abuse with patients coming in and being drunk and abrasive and on a repetitive basis, and now it just happens to be a few patients who come from [a specific First Nation]. And there's just kind of this anger in general… I think we still try to be professional but we still have that anger in us so I know that it wouldn't take much for us to kind of say something or to feel differently. I don't know that we necessarily act on it but I do think that we feel differently towards people from [the nearby First Nation] just based of some experiences that we've had with some people." (N01)<br><br>"And the unfortunate thing is, when you have young nurses who go in [to ED work] who have no experience with First Nations, that's when the racism and the short temperedness comes out, because they don't understand the dynamics of the people." (N10)

**Power Differentials in the ED**	
	"[W]e're people that have the say…you know, they're coming to us so we're the people that have the power...we're the ones who have the power so we're not actually explaining it to them. And sometimes that's just the inability to actually, you know, relay information really well." (N5)
Provider Power to Advocate for Care or not	"a good example, I think, of a patient I saw… young person in probably his 20s, who was visibl... well I mean you can never know for sure, but visibly First Nations, or at least that's my recollection is that he was First Nations, has come in with trimalleolar fracture. I receive him in hand over, and that's an ankle fracture that is almost always dealt with, it should be dealt with surgically… But he was a patient that used meth for sure, and I think other substances as well…. And was homeless, and so orthopedics was concerned that he would be non-compliant post-surgery. I'm pretty sure, I feel badly saying these things, but I'm pretty sure the surgeon did not want to inherit this patient as a chronic pain patient post-surgery and basically said [the patient] had to prove that he could stay off his ankle for a week, [the surgeon] would see him at follow up and if he could do that then he would do surgery on him… I waited until we had a bed available on our hospital care team and asked them to admit the patient and do some social stabilization, get [an addiction team] involved and at least try to give them a chance to have this surgery." (P13)<br><br>"just as an example, we had a street person who sustained really bad frost bite on his feet. So when we did up his wound care card, one of his orders was 'Feed this client lunch when he comes in, or supper'. And every one of the people that I worked with who didn't like this person went to the manager and said 'I can't believe that they're ordering that we have to feed this person.' Bless my manager. She was into wound care and she defended our order and said, but if you don't want this man admitted to hospital and taking up a bed, and he's coming in every day for his wound care, then the least we can do is feed him because it's the only nutritional value he's going to have in a day and its going to help him heal."(N10)[1]

[1] We would note that in this story, the provider uses the idea that the patient will take up more system resources (through hospital admission) in order to argue that the patient should receive meals – rather than an argument based in patient need or human rights.

**Provider Attitudes towards Care of First Nations Patients**	
Denial of responsibility for 'the past'	"[E]veryone knows about residential school and everyone knows the historical origins of colonialism and the, the poverty and the impact on families. I think everyone knows about it, but actually making that connection and integrating that empathy that should arise from that in the interaction-I think that's very variable." (P11)<br><br>"…its easy for [First Nations people] to say, well residential school this, residential school- if it wasn't for you white men I wouldn't be where I am now. Well, my people weren't here at the time when you guys were put in residential schools. Like we're new immigrants here. So I can't take responsibility for what happened to you, but I can acknowledge it, and I can work with you at healing it because I am acknowledging it." (N10)
Reflections on provider role in wider historical and political context	"I think in general I try to be extra cautious with my First Nations patients about engaging in shared decision making when its applicable. Um, given kind of the history of oppression and colonialism." (P17)<br><br>**"…**you know the old Indian hospitals and the experiences that happened there, and hospitals are a place of authority and 'the government,' the white government… And I'm representing the authority and the institutions that's kind of screwed them over for generations." (P6)<br><br>"I hope that some of the oppressive structure of medicine, some of the Indian hospital, residential school power structure, I hope some of that gets disrupted when patients who are Indigenous see me." (P7)<br><br>"I think one of the big challenges that we have in healthcare is a lack of understanding about trauma-informed care and the actual social implications, the physical implications, emotional, mental, all of those pieces, [including] spiritual [implications]. I think health care providers in general, there's a big gap in terms of we need to have a lens of trauma informed care and the generational trauma and pieces that are definitely present within First Nations communities." (N2)

Provider Efforts to Address Racism and Systemic Issues in First Nations Care	
Efforts to mitigate past negative experience with healthcare	"With most of my patients I just presume to call them by their first name. I never do that with my Indigenous patients. Just because, you know, I just sort of want to… be, I don't know, a bit sort of explicit, or conscious, or external about the idea that I'm treating them with respect." (P9)<br><br>"I'll often work in a detail to a clinical case that I'm signing over to another colleague to try to mitigate potential biases. So you know, if its an older patient, you know, I'll often describe them, if its appropriate, as a well-respected elder with multiple grandchildren, and just to sort of add more human dignity and sympathy to the case than I would have potentially done for a… white person." (P11)
Efforts to mitigate systemic barriers to care	"And I found that patients' specialist follow up appointments get canceled because of transportation issues and then they get like banned from that specialist's office. They're like 'don't refer to us again. They had too many no shows.' And its like 'poor old guy,' he like doesn't speak English. The van broke down, they couldn't get there. Like, they're not trying to waste your time… So I just try to really advocate for patients to say, listen like we're going to try to set it up in a way as best as we can for you, within a system that doesn't cater to you, and then we'll try to get everything around you that we can, like gas money, medical transport van, translator, like whatever we need to have to set you in order for this to be successful." (P7)<br><br>"I don't mind people coming back [to the ED for follow up care]. And especially the patient population that I was really flexible with that was kids. 'Why don't you bring your kid back tomorrow and I'll just check on them and make sure they're ok.' You know, I know I'll be post call, just bring your kid in and I'll just check on them. And I would tell them make sure you check in with me, don't check into the regular emergency department. 'Check in,' this works better in rural [hospitals], 'check in and say you're seeing me, I'll have your chart and we'll just check on you if you're here tomorrow." (P7)<br><br>"…we might say it's kind of unrealistic for them to drive home, have an hour's sleep and then turn around and come back. So we might say, 'look we've got some empty beds that we've closed, we're not staffed for, so you guys can just sleep here overnight." (N5)

	"But sometimes these people [First Nations patients] don't have phones, they don't have a ride, they have no way to get to that follow up appointment. So sometimes we try to get transportation to bring them back at eight in the morning and then we get an ambulance arranged to send them to [the larger hospital] which isn't a great way to use resources." (N8)
