# Supplementary material for: Impacts of racism on First Nations patients' emergency care: results of a thematic analysis of healthcare provider interviews in Alberta, Canada
Source: BMC Health Serv Res. 2022 Jun 21;22:804. doi: 10.1186/s12913-022-08129-5 (PMC9210059; doi:10.1186/s12913-022-08129-5)
Supplement: Supplementary file 2 — Additional file 2. [file 12913_2022_8129_MOESM2_ESM.pdf]

Supplement 1: Qualitative Sampling Frame

	Physicians	Nurses
Remote	4 (may include Nurse Practitioners)	4 (at nursing stations)
Rural	4	4
Urban	4	4

*Strive for a balance of male and female participants overall, as well as inclusion of self-identified First Nations participants.

Supplement 2: First Nations Emergency Department Quality of Care Project Clinician Interview Questions

Overall, the interviewer should pick up on themes of history, biases, stereotypes, generalizations, trust, communication, differences in power between patients and providers, distinct roles of physicians and nurses and how these roles impact how they can deliver care for FN patients.

1. Do you feel patients' expectations for care are met in the emergency department?
  - Prompt for specific examples of why or why not.

What do you feel patients' expectations for care are in the ED?

2. Do you feel First Nations patients' expectations for care are met in the emergency department?
  - Prompt for specific examples of why or why not, prompt for differences from non-FN patients.

Prompt for expectations at different phases such as triage, interview/testing, discharge, follow-up.

3. Do you feel patients receive appropriate care in the emergency department?
  - Prompt for specific examples of why or why not.

4. Do you feel First Nations patients receive appropriate care in the emergency department?
  - Prompt for specific examples of why or why not, prompt for differences from non-FN patients.

- Prompt for expectations at different phases such as triage, interview/testing, discharge, follow-up.

5. When there are multiple care options, are patients part of the decision making about their care in the emergency department?
   - Prompt for how this works, what kind of things do they get to decide?

6. Are First Nations patients part of the decision making about their emergency department care?
   - Prompt for how this works, what kind of things do they get to decide?
   - Do you feel First Nations patients are invited to participate in decision making to the same degree as other patients?
   - Do you perceive any difference in First Nations patients' engagement in decision making?

7. Are patients involved in the decision making about their care plan and recovery after leaving the emergency department?
   - Prompt for how this works, what kind of things do they get to decide. How do differences in availability of care options impact after-care decisions?
   - If the provider mentions things they to address barriers to follow up care, prompt for specific details.

8. Are First Nations patients involved in the decision making about their care plan and recovery after leaving the emergency department?
   - Prompt for how this works, what kind of things do they get to decide. How do differences in availability of care options impact after care decisions?
   - If the provider mentions things they do to address barriers to follow up care, prompt for specific details.

9. Have you had cultural safety or sensitivity training about Indigenous peoples?
   - If yes, do you think having this training has benefited your patients?

10. Have you explored cultural differences of those who are different from you?

11. We'd like to explore understandings of racism. How would you define racism?

12. Do you think there are times racism impacts patient experiences in the emergency department?

- Ask for specific examples.
- If the provider mentions stereotypes, ask: What are the stereotypes or pre-expectations around FN patients that you see or hear?
- Have you seen those stereotypes impact how a patient is treated? Can you give an example?

13. What does reconciliation mean to you?

14. What would reconciliation look like in the emergency department?

For this project we analyzed Alberta Health Services data on Emergency Department visits from 2012 to 2017 to compare differences in the numbers and reasons for ED visits between First Nations and non-First Nations people, and to look at some quality of care indicators.

15. We found that First Nations people live further from the ED than non-First Nations people, and that more First Nations people arrive by ambulance. Is this consistent with your experience and knowledge, and do you have any thoughts as to the reasons for these findings?

16. We also found that significantly more FN people leave without being seen by a doctor, and leave more before discharge. Is this consistent with your own experience and how might you explain these findings?

17. The data shows that more FN visits are triaged as less acute. Is this consistent with your own experience? Can you explain why this may be?
